# Survival benefit of induction chemotherapy for locally advanced nasopharyngeal carcinoma: prognosis based on a new risk estimation model

**DOI:** 10.1186/s12885-021-08381-8

**Published:** 2021-05-29

**Authors:** Wei Liu, Bolong Yu, Yunfan Luo, Junzheng Li, Xiaofei Yuan, Shuting Wu, Bijun Liang, Zehong Lv, Yanfei Li, Xinyu Peng, Juan Lu, Xiaohong Peng, Xiong Liu

**Affiliations:** 1 Department of Otolaryngology-Head and Neck Surgery, Nanfang Hospital, Southern Medical University, Guangzhou, 510515 Guangdong PR China; 2 Department of Otolaryngology-Head and Neck Surgery, Guangzhou Red Cross Hospital, Medical College, Jinan University, Guangzhou, 510220 Guangdong PR China

**Keywords:** Nasopharyngeal carcinoma, Chemoradiotherapy, Risk estimation model

## Abstract

**Background:**

Although the National Comprehensive Cancer Network (NCCN) Guidelines recommend CCRT+AC and IC + CCRT as level 2A evidence for treatment of the locoregionally advanced NPC (II-IVa), IC + CCRT+AC could also be an alternative but it is seldom used because of the low completion rates. This article aimed to compare the effectiveness of the three radiotherapy regimens using a large-scale retrospective study.

**Methods:**

This retrospective single center analysis enrolled 1812 diagnosed NPC patients at Nanfang Hospital from January 2005 to December 2015 and only 729 patients met the inclusion criteria and were analyzed. Patients without distant metastasis, age of 18–70 years, Karnofsky scores of at least 70,stage III-IVb, and adequate adequate bone marrow, liver and renal function. Were enrolled. Adverse events and other categorical variables were compared by Pearson chi-square test or Fishier exact test. Time-to-event data were described with the Kaplan-Meier curves, time-to-event intervals compared with the log-rank test. We did multivariable analyses with the Cox proportional hazards model to test the independent signifi cance of diff erent factors. Cox proportional hazards model was used to estimate the β regression coeffi cient, *p* value, and hazard ratio and its 95% CI for each of the selected risk predictors.

**Results:**

The median follow-up time was 47 months. Kaplan-Meier analyses revealed no significant differences among three groups in 3-year failure-free survival (FFS, *P* = 0.225), 3-year overall survival (OS, *P* = 0.992), 3-year locoregional failure-free survival (LFFS, *P* = 0.549), and 3-year distant failure-free survival (DFFS, *P* = 0.174). Stratified survival analysis based on the risk scoring model revealed no differences in FFS, OS, LFFS, and DFFS between IC + CCRT and CCRT+AC groups for low-risk patients, however, the 3-year OS (88.3% vs. 77.6%, *P* = 0.049) and 3-year DFFS (84.0% vs.66.8%, *P* = 0.032) were respectively significantly better in IC + CCRT group compared with CCRT+AC group for high-risk patients.

**Conclusions:**

Compared with CCRT+AC, IC + CCRT lowers distant metastasis rate and improves OS among patients with locally advanced NPC in high risk group.

**Supplementary Information:**

The online version contains supplementary material available at 10.1186/s12885-021-08381-8.

## Background

Nasopharyngeal carcinoma (NPC) is a type of head and neck cancer rare throughout most of the world but common in specific geographic areas, such as Southern China. According to the International Agency for Research on Cancer, there were 129,079 new cases of NPC and 72,987 deaths from it in 2018 worldwide in 185 countries, with approximately 70% of new cases occurring in Eastern and Southeastern Asia [1]. Approximately 70% of newly diagnosed NPC patients present with stage III or IV disease due to difficulties in early detection and high incidences of locoregional metastasis and distant metastasis [2]. The platinum-based concurrent chemoradiotherapy (CCRT) regimen is recommended by the NCCN Guidelines for advanced stage NPC patients [2–4], but almost 30% of NPC patients are prone to suffer from locoregional recurrence or distant metastases and experience treatment failure [2, 3, 5]. Therefore, the addition of induction chemotherapy (IC) or adjuvant chemotherapy (AC) to CCRT have been widely accepted with the rationale of improving distant control in the clinical practices. There are three main ways of combining systemic chemotherapy and CCRT with respect to the time sequences of each modality, including IC + CCRT, CCRT+AC and IC + CCRT+AC. However, the most effective regimen remains controversial.

Although the survival benefit of IC + CCRT regimen remains controversial [6–9], several clinical trials have reported that IC + CCRT regimen provided better survival benefit compared to CCRT alone [6–9]. In addition, two recent large multicenter phase III clinical trials showed that IC significantly improved the 3-year failure-free survival rate (FFS), distant failure-free survival rate (DFFS) and overall survival rate (OS) [10, 11]. Another multicenter, randomized, controlled, phase 3 clinical trial found that addition of IC to CCRT significantly improved recurrence-free survival and overall survival for patients with locoregionally advanced NPC [12]. Therefore, IC + CCRT regimen was changed from a category 3 to a category 2A recommendation in the latest version of NCCN Guidelines (2020.V2).

It is still uncertain whether NPC patients might benefit from additional AC after CCRT. Several clinical studies have confirmed the effectiveness of CCRT+AC [13–15], and recent meta-analyses also concluded that the CCRT+AC regimen could improve OS, PFS, LFFS, and DFFS compared to CCRT alone [16–18]. However, a randomized controlled trial (RCT) reported by Chen et al. found that CCRT+AC failed to significantly improve LFFS of patients with locoregionally advanced NPC compared to CCRT alone (86% vs. 84%, *P* = 0.13) [19], which were similar to the data of Kwong et al. [20]. Therefore, CCRT+AC regimen was changed from a category 1 to a category 2A recommendation in the 2014 NCCN Guidelines, and IC + CCRT+AC regimen was not recommended by NCCN Guidelines due to the serious chemotherapy-induced adverse events. However the efficacy analysis of treatment regimens in the above studies was not based on a more refined patient stratification, which may lead to a biased result.

At present, both CCRT+AC and IC + CCRT are recommended as category 2A by the NCCN Guidelines for advanced stage NPC patients, but the most effective regimen for integrating chemotherapy with CCRT are still unknown yet. Thus, we performed a retrospective study to compare survival benefit and toxicities of CCRT+AC, IC + CCRT and IC + CCRT+AC regimens.

A risk scoring model is a statistical method that uses statistical methods to analyze the risk factors associated with a patient developing a disease or producing an adverse outcome and generates a risk score, which is used to predict the risk of the disease. A risk scoring model is now widely used in patient management. Risk scores are also used for patient counseling, management, clinical diagnosis, risk stratification, treatment selection, and prognosis prediction. The method has been widely used in the field of cardiovascular disease [21, 22] and more recently it has been used to predict the prognosis of liver disease [23–25]. While the successful development and use of cardiovascular and liver disease risk scores has demonstrated the benefits of this approach to patients and physicians, there is no standardized and accurate guideline regarding the prognostic risk assessment of patients with locally advanced nasopharyngeal carcinoma. For patients with locally advanced nasopharyngeal carcinoma, the ability to determine the risk of post-treatment failure prior to treatment would enable clinicians to determine treatment decisions and allocate health resources more rationally at an early stage, and a stratified survival analysis under different risk strata might provide new ideas for the selection of treatment options. Based on the previous reports, we established a new risk scoring model with the baseline clinical variables to predict the prognosis of patients with locally advanced nasopharyngeal treated with different regimens.

## Methods

### Patients

**Fig. 1:**
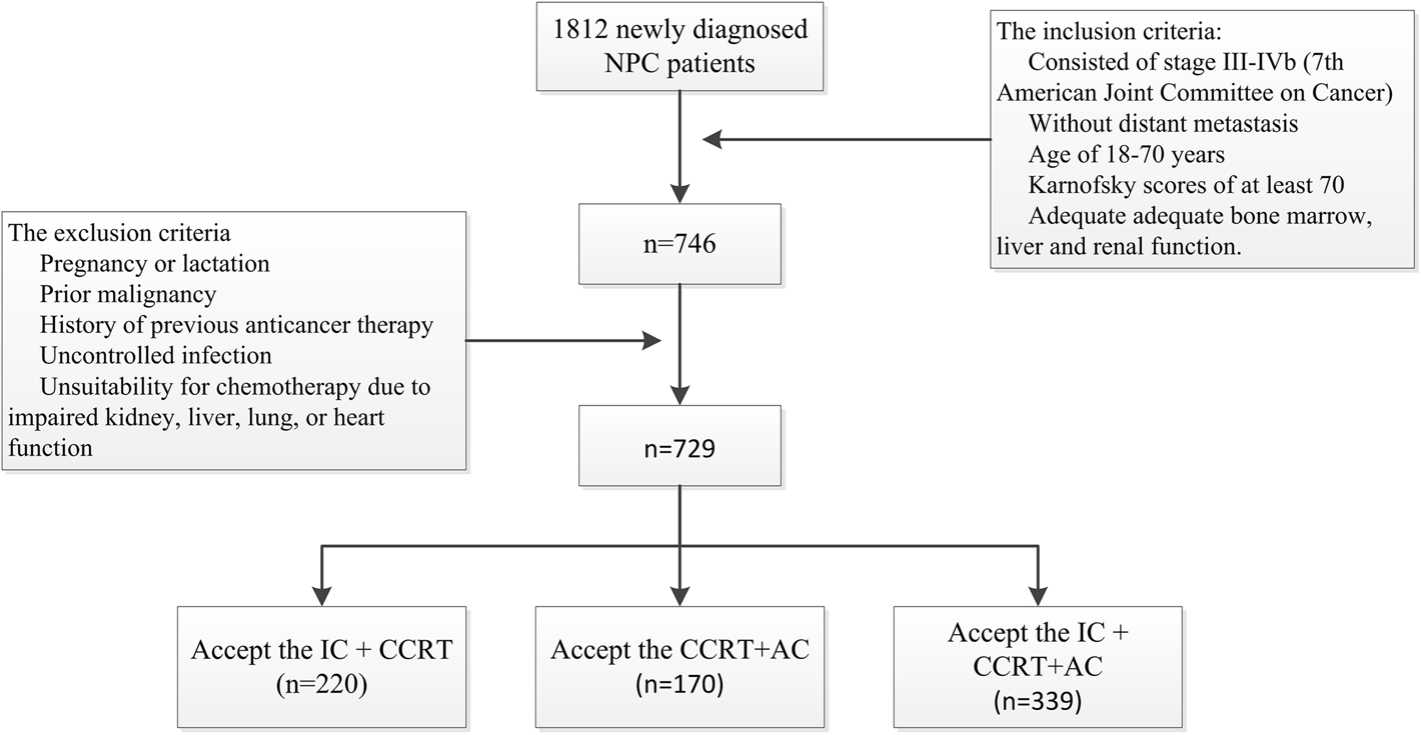
The diagram of enrolled patients with nasopharyngeal carcinoma patients. CCRT = concurrent chemoradiotherapy

From January 2005 to December 2015, 1812 newly diagnosed NPC patients from Nanfang Hospital were enrolled in this study (Fig. 1). The inclusion criteria consisted of stage III-IVb in 7th Edition of the American Joint Committee on Cancer (7th AJCC) without distant metastasis, age of 18–70 years, Karnofsky scores of at least 70, and adequate adequate bone marrow, liver and renal function. The exclusion criteria consisted of pregnancy or lactation, prior malignancy, history of previous anticancer therapy, uncontrolled infection, and unsuitability for chemotherapy due to impaired kidney, liver, lung, or heart function. Out of 1812 patients, we deleted 1083 patients who did not meet the research criteria, including 432 patients with early stage I-II; 163 cases have been identified or uncertain distant metastasis; 1 lactating patient; 9 patients with a history of malignant tumors; 7 patients with 3-dimensional conformal therapy; 9 patients younger than 18 years old; 45 patients older than 70 years old; 835 receiving other treatments Modalities (such as CCRT or radiation therapy). Finally, 729 patients were included. A pretreatment evaluation of patients is described in Fig. 1. Our protocol was approved by the Ethics Committee of Nanfang Hospital of Southern Medical University (NFEC-2017-165).

### Chemotherapy

The interval between platinum chemotherapy cycles is 21 days. Induction chemotherapy and adjuvant chemotherapy include TP, TPF, and PF. Among them, the TP regimen is docetaxel 60 mg/m2/d or paclitaxel 135 mg/m2/d on day 1 and cisplatin 25 mg/m2/d on days 1 to 3, TPF is docetaxel 60 mg/m2/d or paclitaxel 135 mg/m2/d on day 1, cisplatin 25 mg/m2/d on days 1 to 3, and 5-fluorouracil 600 mg/m2/d on days 1 to 5 and PF is cisplatin 25 mg/m2/d on days 1 to 3 and 5-fluorouracil 600 mg/m2/d on days 1 to 5). The concurrent chemotherapy regimens (1 or 2 cycles) included cisplatin monotherapy (once every 3 weeks, 25 mg/m2/d on days 1 to 3) and TP (docetaxel 60 mg/m2/d or paclitaxel 135 mg/m2/d on day 1 and cisplatin 25 mg/m2/d on days 1 to 3).

### Radiotherapy

All patients received intensity-modulated radiotherapy 5 times a week for 6–7 weeks, with 2.12–2.24 Gy each time. The cumulative radiation dose to the primary tumor is 66–70 Gy, and the cumulative radiation dose to the affected area of the neck is 60–66 Gy. Irradiate all possible local infiltration sites and bilateral cervical lymphatics with a dose of 50–54 Gy. The radiation dose of each organ is the anterior nasal area (6–8 Gy), the parapharyngeal area (6–8 Gy) or the skull base area (6–8 Gy).

### Follow-up

In the 2 years after the end of treatment, the patients will be followed up every 3 months, and then every 6 months from the 3rd year to the 5th year. Each follow-up is carried out in accordance with the follow-up principles for common nasopharyngeal carcinoma: physical examination; electronic nasopharyngeal fiberscope; bone imaging (radiation computed tomography); chest X-ray; nasopharyngeal neck MRI; abdominal ultrasound and biopsy of suspected parts or a needle biopsy to confirm the nature of the pathology. The related side effects were evaluated according to the Common Terminology Standard for Adverse Events (CTCAE version 4.0). At the 16th week after the end of treatment, the efficacy was evaluated according to the solid tumor response evaluation criteria (version 1.1). The primary endpoint of the study is failure-free survival (FFS), and the secondary endpoints are overall survival (OS), long-distance failure-free survival (DFFS) and local area failure-free survival (LFFS). We calculated the OS from the initial pathological diagnosis to death. At the same time, we also calculated the FFS from the date of initial pathological diagnosis to the date of treatment failure or death from any cause (whichever comes first). For local and long-distance failure-free survival analysis, we recorded the latency of the first local or long-distance failure (that is, the time from the pathological diagnosis).

### Selection of risk factors and construction of the risk scoring model

The risk factors selected for the derivation of the risk scoring model were based on previous literature [26–28], and the risk factors ultimately identified for inclusion were those previously shown to be strongly associated with nasopharyngeal carcinoma prognosis. These risk factors which are clinically common and readily available, include: plasma EBV-DNA copy number, sex, age and smoking.

The risk scoring model was developed in 3 steps. Firstly, the regression coefficient, *P* value, and risk ratio of treatment failure were estimated by the Cox proportional hazards model. Next, the regression coefficient of each risk predictor in the Cox proportional hazard model was divided by the regression coefficient of smoking. The obtained value was multiplied by 5 and rounded into an integer value to generate the risk score and then add up the risk scores of all risk factors and calculate the median risk score. Finally, all patients were divided into the high-risk and low-risk groups according to the median risk score:patients with a cumulative risk score greater than median risk score were assigned to the high-risk group, while patients with a cumulative risk score less than or equal to median risk score were assigned to the low-risk group.

### Statistical analysis

All statistical analyses were done with SPSS 23.0 (SPSS Inc., Chicago, IL). Adverse events and other categorical variables among IC + CCRT, CCRT+AC, and IC + CCRT+AC groups were compared by Pearson chi-square test. Time-to-event data were described with the Kaplan-Meier curves, time-to-event intervals compared with the log-rank test. We did multivariable analyses with the Cox proportional hazards model to test the independent signifi cance of diff erent factors. Cox proportional hazards model was used to estimate the β regression coeffi cient, *p* value, and hazard ratio and its 95% CI for each of the selected risk predictors.

## Results

### Patient characteristics

Total 1812 NPC patients from Nanfang Hospital were enrolled in this study. The inclusion and exclusion criteria are shown in Fig. 1 and only 729 patients met the inclusion criteria and were analyzed (220 patients in IC + CCRT group, 170 in CCRT+AC group, and 339 in IC + CCRT+AC group). The median follow-up time for entire cohort was 47 months (range,1–135 months). Pretreatment patient characteristics stratified by treatment modalities were listed in Table 1. With the exception of age, clinical stage and T category, no significant differences in baseline demographic and clinical characteristics were found among the 3 treatment groups.

**Table 1:** Clinical characteristics of nasopharyngeal carcinoma patients

	IC + CCRT(***n*** = 220)	CCRT + AC (***n*** = 170)	IC + CCRT + AC(***n*** = 339)	***P*** value
**Sex**				
**Male**	157 (71.4%)	128 (75.3%)	263 (77.6%)	0.251
**Female**	63 (28.6%)	42 (24.7%)	76 (22.4%)	
**Age group, years**				
≤ 55	164 a (74.5%)	141 a,b (82.9%)	296 b (87.3%)	0.001
> 55	56 a (25.5%)	29 a,b (17.1%)	43 b (12.7%)	
**Staging**				
III	85 a (38.6%)	88 b(51.8%)	135 a (39.8%)	0.033
IVA	112 a (50.9%)	61 b (35.9%)	162 a (47.8%)	
IVB	23 a(10.5%)	21 a(12.4%)	42 a(12.4%)	
**EBV-DNA levels**				
≤ 1500	97 (54.8%)	51 (46.8%)	122 (51.3%)	0.418
> 1500	80 (45.2%)	58 (53.2%)	116 (48.7%)	
**Smoking**				
Yes	91 (45.5%)	61 (37.9%)	137 (42.9%)	0.339
No	109 (54.5%)	100 (62.1%)	182 (57.1%)	
**Tumor category**				
1	22 a (10.0%)	21 a (12.4%)	42 a (12.4%)	0.040
2	28 a (12.7%)	40 b (23.5%)	46 a (13.6%)	
3	54 a (24.5%)	39 a (22.9%)	75 a (22.1%)	
4	116 a (52.7%)	70 a (41.2%)	176 a (51.9%)	
**Node category**				
0	12 (5.5%)	11 (6.5%)	20 (5.9%)	0.738
1	54 (24.5%)	43 (25.3%)	66 (19.5%)	
2	127 (57.7%)	94 (55.3%)	210 (61.9%)	
3	27 (12.3%)	22 (12.9%)	43 (12.7%)	

The total percentage may not add up to100% due to rounding. The clinical stages were evaluated according to the 7th edition of the American Joint Committee on Cancer TNM staging system. Data were expressed as n (%); *P* value was calculated by chi-square test. Each subscript letter denotes a subset of treatment categories whose column proportions do not differ significantly from each other at the 0.05 level

### Toxicity

There were significant differences in the incidence of grade 3–4 leukopenia among three treatment group, which was the most common adverse reactions in blood and lymphatic system during chemotherapy. Further analysis demonstrated that the incidence rate of grade 3–4 leukopenia was significantly lower in IC + CCRT group than those of CCRT+AC and IC + CCRT+AC groups (16.4% vs. 31.8 and 28.8% respectively, both *P* < 0.01), but no significant difference was found between CCRT+AC group and IC + CCRT+AC group (*P* = 0.49) (Table 2). Additionally, there was no significant difference in the incidence of non-hematological adverse events among three treatment groups, including grade 2–3 gastrointestinal reactions (33.9% vs. 40.2% vs. 30.7%, *P =* 0.100), grade 3–4 oral mucositis (16.3% vs. 15.3% vs. 20.0%, *P* = 0.332) and grade 3–4 radiation-induced dermatitis (15.1% vs. 15.9% vs. 17.4%, *P* = 0.742) (Table 2).

**Table 2:** Cumulative adverse events during treatment by maximum grade per patient

	IC + CCRT(***n*** = 220)	CCRT + AC (***n*** = 170)	IC + CCRT + AC(***n*** = 339)	***P*** value
**Leukopenia (Grade 3–4)**	36a (16.4%)	54b (31.8%)	97 b(28.8%)	0.001
**Skin reaction (Grade 3–4)**	33a (15.1%)	27a (15.9%)	59a (17.4%)	0.742
**Mucositis (Grade 3–4)**	33a (16.3%)	26 a(15.3%)	67 a(20.0%)	0.332
**Nausea or vomiting (Grade 2–3)**	74 a(33.9%)	68a (40.2%)	103 a(30.7%)	0.100

Grade 3–4 leukopenia, mucositis, and skin reactions, and grade 2–3 gastrointestinal reactions were recorded according to Common Terminology Criteria for Adverse Events (CTCAE) Version 4.0. Data were expressed as n or n(%); *P* value was calculated by chi-square test. Each subscript letter denotes a subset of treatment categories whose column proportions do not differ significantly from each other at the 0.05 level

### Survival

Overall, treatment failure occurred in 212 of 729 patients (29.1%) (56 of 220 patients (25.5%) in the IC group, 44 of 170 patients (25.9%) in the AC group, and 112 of 339 patients (33.0%) in the IC + CCRT+AC group. The pattern of treatment failure was not significant different across the three treatment groups (Table 3).

**Table 3:** Number of events in each group

	IC + CCRT(***n*** = 220)	CCRT + AC (***n*** = 170)	IC + CCRT + AC(***n*** = 339)	***P*** value
Failures	56 (25.5%)	44 (25.9%)	11 (33.0%)	*P* = 0.225
Deaths	25 (11.4%)	22 (12.9%)	44 (13.0%)	*P* = 0.992
Locoregional failure	21 (9.5%)	13 (7.6%)	36 (10.6%)	*P* = 0.549
Distant failures	33 (15.0%)	33 (19.4%)	78 (23.0%)	*P* = 0.174

Data are n (%) or rate (95% CI). *CCRT* Concurrent chemoradiotherapy. *P* values were calculated with the log-rank test

**Fig. 2:**
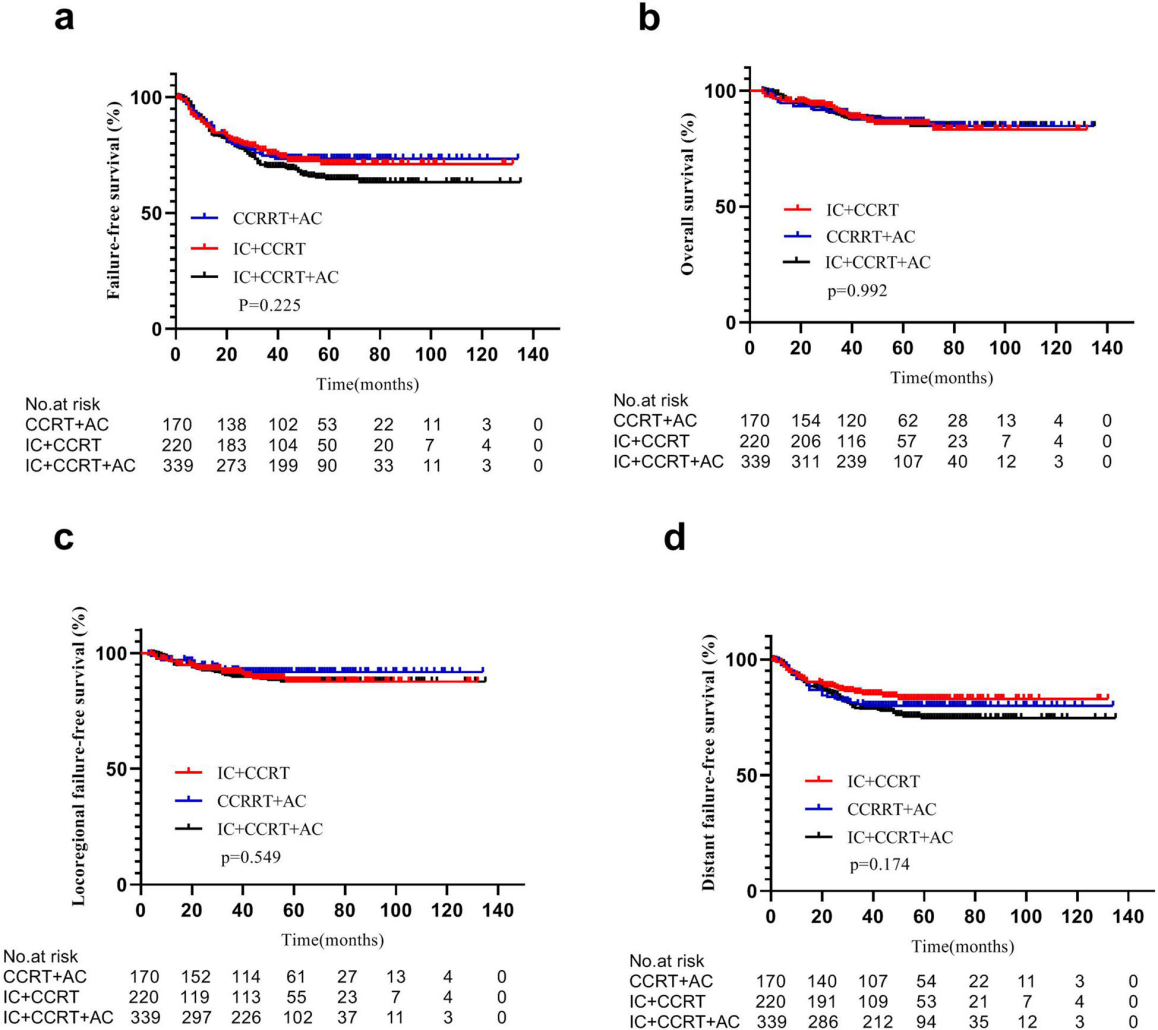
Kaplan–Meier survival curves of IC + CCRT, CCRT+AC, and IC + CCRT+AC treatment groups. There were no differences in survival outcomes among treatment groups (*P* > 0.05 by rank-sum test). The primary endpoint is failure-free survival (FFS) rate, defined as the time between the initial pathological diagnosis of nasopharyngeal carcinoma (NPC) and treatment failure or final follow-up. Secondary endpoints include overall survival (OS), locoregional failure-free survival (LFFS), and distant failure-free survival (DFFS). IC = induction chemotherapy. AC = adjuvant chemotherapy. CCRT = concurrent chemoradiotherapy

FFS (75.6% vs.74.8% vs.69.9%, *P* = 0.225), OS (90.1% vs. 90.4% vs. 88.9%, *P* = 0.992), LFFS (91.7% vs.92.5% vs.89.9%, *P* = 0.549) or DFFS (84.9% vs.80.6% vs.78.7%, *P* = 0.174) did not differ significantly between the IC + CCRT, CCRT+AC, and IC + CCRT+AC groups (Fig. 2, Table 3). The IC + CCRT+AC regimen provided no additional survival benefit.

On multivariable analysis, we found that treatment group was not a significant predictive factor for FFS, OS, LFFS, and DFFS. However, age acted as an independent prognostic factor for FFS (HR 1.479, 95%CI 1.008–2.169, *P* = 0.046), OS (HR 2.402, 95%CI 1.425–4.049, *P* = 0.001), and LFFS (HR 1.984, 95%CI 1.062–3.708, *P* = 0.032). Clinical stage was also an independent prognostic factor for FFS (stage IVb vs. III: HR 1.81, 95%CI 1.095–2.992, *P* = 0.021) and OS (stage (IVa vs. III: HR 1.857, 95%CI 1.037–3.326, *P* = 0.037). EBV-DNA copy number was an independent prognostic factor for FFS (HR 1.835, 95%CI 1.289–2.613, *P* = 0.001) and DFFS (HR 1.955, 95%CI 1.263–3.027, *P* = 0.003). Finally, smoking was an independent prognostic factor for OS (HR 1.922, 95%CI 1.035–3.568, *P* = 0.038) (Supplementary Table 1, 2, 3 and 4).

### Risk scoring model and stratified analyses

The regression coefficient β of the multivariate Cox proportional hazards regression model and the risk scores to predict treatment failure were illustrated in Table 4. Patients with complete follow-up (*n* = 488) were stratified into a high-risk group (*n* = 214) of treatment failure with risk scores > 34 and a low-risk group (*n* = 274) of treatment failure with scores ≤34. For example, a female patient (risk score = 0) aged 33 years old (risk score = 0) in clinical stage IVa (risk score = 12) with smoking history (risk score = 5) and plasma EBV-DNA copy number 3600 (risk score = 22) would have a cumulative risk score of 39, and that belongs to the high-risk group. The results of survival analysis indicated that IC + CCRT+AC regimen failed to improve survival rate, and additional cycles did not provide further survival benefit. Therefore, only IC + CCRT and CCRT+AC groups were subjected to stratified survival analysis based on the risk score.

**Table 4:** Estimation of hazard ratio, β coefficient, and risk score based on a multivariate Cox proportional risk model

	HR	β coefficient	***P*** value	Risk score
**Sex**				
**Female**	1	1		0
**Male**	1.406 (0.85–2.324)	0.34	0.185	12
**Age group, years**				
**≤ 55**	1	1		0
**> 55**	1.414 (0.973–2.055)	0.346	0.069	12
**Staging**				
**III**	1	1	..	0
**IVA**	1.396 (0.958–2.036)	0.334	0.083	12
**IVB**	1.793 (1.087–2.958)	0.584	0.022	21
**Smoking**				
**No**	1	1		0
**Yes**	1.150 (0.792–1.670)	0.14	0.463	5
**EBV-DNA levels**				
**≤ 1500**	1	1		0
**> 1500**	1.848 (1.297–2.633)	0.614	0.001	22

**Fig. 3:**
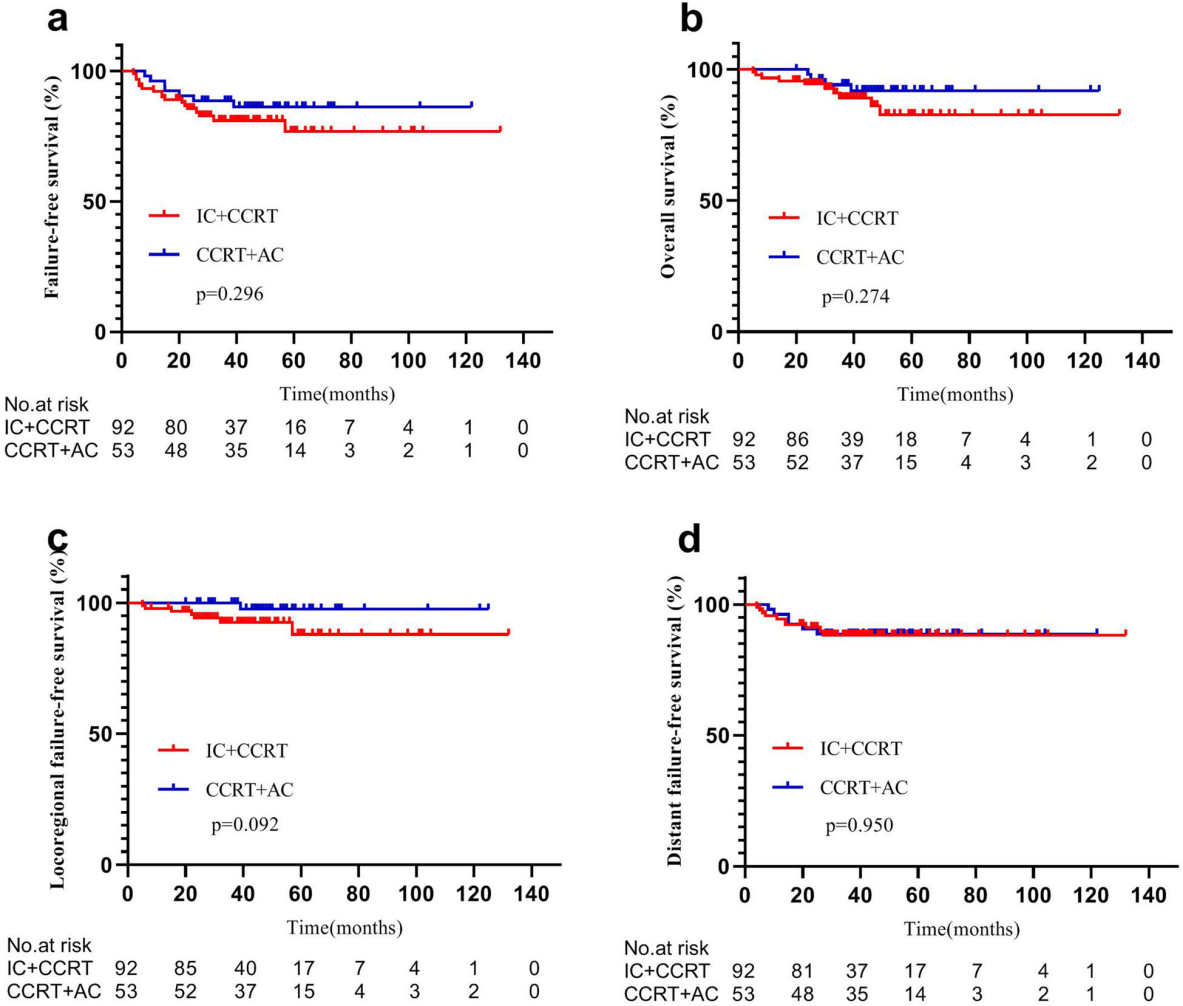
Kaplan–Meier survival curves of IC + CCRT and CCRT+AC patients classified as low risk for treatment failure according to a novel risk scoring system. There were no significant differences in survival endpoints (*P* > 0.05 by rank-sum test). IC = induction chemotherapy. AC = adjuvant chemotherapy

**Fig. 4:**
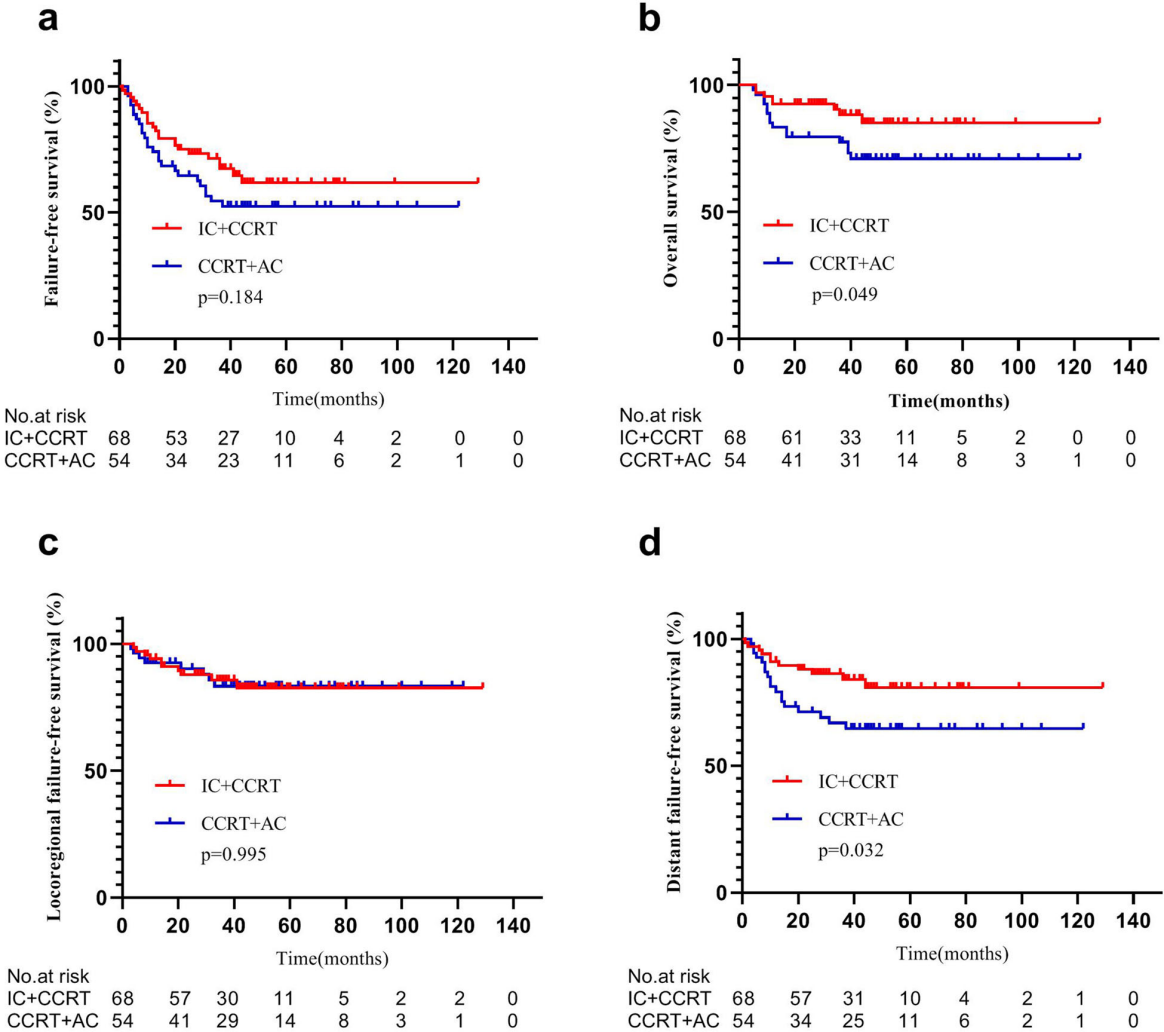
Kaplan–Meier survival curves of IC + CCRT and CCRT+AC patients classified as high risk for treatment failure according to a novel risk scoring system. Patients in the IC + CCRT group achieved better 3-year overall survival (OS) and 3-year distant failure-free survival (DFFS) (both *P* < 0.05 by rank-sum test). IC = induction chemotherapy. AC = adjuvant chemotherapy

The results indicated no significant difference between low-risk patients of IC + CCRT and CCRT+AC groups in 3-year FFS (81.1% vs. 88.6%, *P* = 0.296), OS (89.2% vs. 94.2%, *P* = 0.274), LFFS (92.6% vs. 100%, *P* = 0.092), and DFFS (88.2% vs. 88.6%, *P* = 0.950) (Fig. 3). In contrast, IC + CCRT regimen improved 3-year OS (88.3% vs. 77.6%, *P* = 0.049) and 3-year DFFS (84.0% vs. 66.8%, *P* = 0.032) compared with CCRT+AC regimen in high-risk patients, but 3-year FFS (67.3% vs. 54.5%, *P* = 0.184) and 3-year LFFS (85.7% vs. 83.3%, *P* = 0.995) did not show any significant difference in high-risk patients between IC + CCRT and CCRT+AC groups (Fig. 4).

## Discussion

In this retrospective study, the survival benefit and safety of IC + CCRT, CCRT+AC, and IC + CCRT+AC regimens were compared for locoregionally advanced NPC. The incidence of grade 3–4 acute leukopenia was significantly higher in patients of IC + CCRT+AC and CCRT+AC groups than those in IC + CCRT group, likely due in part to a reduced tolerance following CCRT. Almost every patient suffered from radiation-induced oropharynx and hypopharynx mucositis during CCRT, which led to insufficient food intake and poor nutritional status. In addition, bodily functions tend to decline after CCRT, resulting in higher incidence of hematologic toxicity. In contrast, the incidence of grade 3–4 oral mucositis and radiodermatitis had no significant difference among the three treatment groups, possibly because oral mucositis and radiation-induced dermatitis depended mainly on the type and amount of radiation used rather than the timing of chemotherapy.

Compared with IC + CCRT, the proportion of young patients in the IC + CCRT + AC group is higher. The possible reason is that the IC + CCRT + AC group is clinically considered to have greater side effects, so that it is difficult for older patients to use this program. There are also differences in the patient proportion of T staging among the three treatment groups. Such differences might not be ideal for statistical comparisons. Younger patient generally mean better survival outcomes. However, we did not observe significant differences with regard to OS, LFFS and DFFS among IC + CCRT, CCRT+AC and IC + CCRT+AC groups. Notably, comparing to IC + CCRT and CCRT+AC, IC + CCRT+AC provided no additional survival benefits,probably because IC + CCRT or CCRT+ AC alone already meets the therapeutic threshold for this class of chemotherapeutic agents, and the additional dosing cycle may simply increase the therapeutic toxicity and further reduce the general condition of the patient and thus the survival benefit of the patient.

The traditional TNM staging system is used as a guide for treatment and prognosis of malignant tumors, but the TNM classification has some limitations. Due to individual differences and tumor heterogeneity, the same treatment regimen might produce quite different survival rate in patients with the same TNM stage. Therefore, a stratified analysis should be used to make the estimate of a treatment effect. Sun et al. found that AC could significantly improve 5-year OS (71% vs. 51%, *P* < 0.01) and 5-year distant metastasis-free survival (80% vs. 54%, *P* = 0.017) in advanced N-stage NPC patients compared to concurrent chemotherapy alone [29]. Twu et al. reported that AC could reduce the incidence of distant metastasis and improve the OS of NPC patients with continuously detectable EBV DNA after radiochemotherapy [30]. However, these studies analyzed only one risk factor that might influence the prognosis of locoregionally advanced NPC. In 2017, Liang et al. performed a retrospective study, divided patients into high-risk and low-risk groups based on multiple factors, including T category, N category and serum protein level, and found that the patients of high-risk group obtained greater OS benefit from AC compared to CCRT alone [31]. The limitation of this study is to enroll the four risk factors into the risk scoring model with the same wights. In fact, the contribution of every risk factor to the prognosis is different. Until now, there is no standard risk scoring model to estimate a treatment effect for locoregionally advanced NPC.

In this study, we constructed a new risk scoring model to predict treatment failure using baseline clinical variables and other risk factors reported previously. Apart from TNM stage, other variables were also included in our model, such as plasma EBV-DNA copy number, sex, age and smoking. Plasma EBV-DNA is a potential biomarker for early screening, follow-up monitoring, and predicting recurrence and metastasis [26]. Xiao et al found that men were more prone to NPC than women with age between 50 and 60 years, identifying sex and age distribution as independent risk factors for prognosis prediction of NPC [27]. Therefore, age of 55 years was regarded as the threshold value to distinguish between high- and low- risk groups. Additionally, cigarette smoking was a moderate risk factor for NPC, because many compounds of tobacco could interact with EBV through activating the virus to induce and promote NPC development [28].

Stratified survival analysis based on our risk scoring model demonstrated that 3-year FFS, OS, LFFS, and DFFS showed no significant difference between IC + CCRT and CCRT+AC groups, but the patients of CCRT+AC group obtained slightly better LFFS, suggesting that the addition of AC to CCRT could improve local control, similar to a meta-analysis by Ribassin-Majed et al [16]. In contrast, IC + CCRT treatment improved 3-year OS and 3-year DFFS compared to CCRT+AC in high-risk patients, in accord with other studies reporting that IC was superior for controlling distant micro-metastases [32, 33] and two multicenter randomized controlled trials (GZ2008 and GZ2011) in Guangzhou, China [10, 11].

Several limitations should be acknowledged in our study. Firstly, the retrospective study did not include many other risk factors during pretreatment evaluation, for example, serum levels of lactate dehydrogenase (LDH) and C-reactive protein (CRP) were potentially important risk factors affecting the clinical prognosis of NPC patients [34, 35], but were not included in our study. In addition, survival outcomes and acute toxicity were only limited to 3 years. The follow-up time should be extended to at least 5 years for comparison of advanced-stage adverse events in future studies.

We found that survival benefits (OS and DFFS) of IC + CCRT, CCRT+AC, and IC + CCRT+AC regimens were on significant difference for patients with locoregionally advanced NPC. However, the incidence of grade 3–4 acute leukopenia was significantly lower in the IC + CCRT group compared to CCRT+AC and IC + CCRT+AC groups. Regarding the chemotherapy toxicity, IC + CCRT regimen was superior to CCRT+AC regimen for those patients with high risk of treatment failure identified by our risk scoring system.

## Conclusions

In conclusion, according to the risk scoring model, IC + CCRT regimen in high-risk patients with locoregionally advanced NPC showed promising clinical outcomes compared with CCRT+AC regimen. However, these conclusions should be further confirmed in larger-scale, prospective, multicenter randomized controlled trials.

## Supplementary Information

**Additional file 1: Supplementary Table 1** Multivariate analysis of failure-free survival (FFS). **Supplementary Table 2.** Multivariate analysis of overall survival (OS). **Supplementary Table 3.** Multivariate analysis of locoreginal failure free survival (LFFS). **Supplementary Table 4.** Multivariate analysis of distant failure free survival (DFFS).

## Data Availability

The datasets used or analysed during the current study are available from the corresponding author on reasonable request.

## Abbreviations

AC: Adjuvant chemotherapy
AJCC: American Joint Committee on Cancer
CCRT: Concurrent chemoradiotherapy
CTCAE: Common Terminology Criteria for Adverse Events
DFFS: Distant failure-free survival
ECT: Emission computed tomography
FFS: Failure-free survival
GTV: Gross tumor volume
IARC: International Agency for Research on Cancer
IC: Induction chemotherapy
LFFS: Locoregional failure-free survival
NCCN: National Comprehensive Cancer Network
NPC: Nasopharyngeal carcinoma
OS: Overall survival
PTV: Planning target volume
RCT: Randomized Controlled Tria
