# Supplementary material for: Survival benefit of induction chemotherapy for locally advanced nasopharyngeal carcinoma: prognosis based on a new risk estimation model
Source: BMC Cancer. 2021 May 29;21:639. doi: 10.1186/s12885-021-08381-8 (PMC8164787; doi:10.1186/s12885-021-08381-8)
Supplement: Supplementary file 1 — Additional file 1: Supplementary Table 1 Multivariate analysis of failure-free survival (FFS). Supplementary Table 2. Multivariate analysis of overall survival (OS). Supplementary Table 3. Multivariate analysis of locoreginal failure free survival (LFFS). Supplementary Table 4. Multivariate analysis of distant failure free survival (DFFS). [file 12885_2021_8381_MOESM1_ESM.docx]

**Supplementary Table 1 Multivariate analysis of failure-free survival (FFS)**

| **FFS** | **HR (95% CI)** | ***P* value** |
| --- | --- | --- |
| **Sex (male vs. female)** | 1.373 (0.829-2.276) | 0.218 |
| **Age group (elderly age vs. young age)** | 1.479 (1.008-2.169) | 0.046 |
| **CCRT+AC group vs. IC+CCRT group** | 1.155 (0.718-1.856) | 0.552 |
| **IC+CCRT+AC group vs. IC+CCRT group** | 1.229 (0.822-1.840) | 0.315 |
| **CCRT+AC group vs. IC+CCRT+AC group** | 0.939 (0.611-1.444) | 0.775 |
| **Overall stage (IVa vs. III)** | 1.395 (0.954-2.039) | 0.086 |
| **Overall stage (IVb vs. III)** | 1.810 (1.095-2.992) | 0.021 |
| **Overall stage (IVa vs. IVb)** | 0.771 (0.479-1.241) | 0.284 |
| **EBV-DNA levels (≥1500 vs. <1500)** | 1.835 (1.289-2.613) | 0.001 |
| **Smoking (Yes vs. No)** | 1.164 (0.801-1.693) | 0.425 |

*P* value is calculated by Cox proportional hazards model; CI: confidential interval.

**Supplementary Table 2 Multivariate analysis of overall survival (OS)**

| **OS** | **HR (95% CI)** | ***P* value** |
| --- | --- | --- |
| **Gender (male vs. female)** | 0.649 (0.308-1.368) | 0.256 |
| **Age group (elderly age vs. young age)** | 2.402 (1.425-4.049) | 0.001 |
| **CCRT+AC group vs. IC+CCRT group** | 1.670 (0.864-3.230) | 0.127 |
| **IC+CCRT+AC group vs. IC+CCRT group** | 1.152 (0.625-2.123) | 0.650 |
| **CCRT+AC group vs. IC+CCRT+AC group** | 1.450 (0.797-2.638) | 0.224 |
| **Overall stage (IVa vs. III)** | 1.857 (1.037-3.326) | 0.037 |
| **Overall stage (IVb vs. III)** | 2.148 (0.997-4.627) | 0.051 |
| **Overall stage (IVa vs. IVb)** | 0.865 (0.430-1.740) | 0.684 |
| **EBV-DNA levels (≥1500 vs. <1500)** | 1.263 (0.761-2.097) | 0.367 |
| **Smoking (Yes vs. No)** | 1.922(1.035–3.568) | 0.038 |

*P* value was calculated by Cox proportional hazards model; CI: confidential interval.

**Supplementary Table 3 Multivariate analysis of locoreginal failure free survival (LFFS)**

| **LFFS** | **HR (95% CI)** | ***P*-value** |
| --- | --- | --- |
| **Sex (male vs. female)** | 1.489 (0.634-3.498) | 0.360 |
| **Age group (elderly age vs. young age)** | 1.984 (1.062-3.708) | 0.032 |
| **CCRT+AC group vs. IC+CCRT group** | 0.745 (0.327-1.698) | 0.484 |
| **IC+CCRT+AC group vs. IC+CCRT group** | 0.884 (0.457-1.710) | 0.714 |
| **CCRT+AC group vs. IC+CCRT+AC group** | 0.843 (0.381-1.866) | 0.674 |
| **Overall stage (IVa vs. III)** | 1.658 (0.849-3.241) | 0.139 |
| **Overall stage (IVb vs. III)** | 2.042 (0.833-5.006) | 0.119 |
| **Overall stage (IVa vs. IVb)** | 0.812 (0.357-1.850) | 0.620 |
| **EBV-DNA levels (≥1500 vs. <1500))** | 1.458 (0.798-2.663) | 0.221 |
| **Smoking (Yes vs. No)** | 0.957 (0.504-1.816) | 0.893 |

*P* value was calculated by Cox proportional hazards model; CI: confidential interval.

**Supplementary Table 4 Multivariate analysis of distant failure free survival (DFFS)**

| **DFFS** | **HR (95% CI)** | ***P*-value** |
| --- | --- | --- |
| **Sex (male vs. female)** | 1.327 (0.711-2.477) | 0.374 |
| **Age group (elderly age vs.young age)** | 1.205 (0.736-1.973) | 0.459 |
| **CCRT+AC group vs. IC+CCRT group** | 1.619 (0.892-2.939) | 0.113 |
| **IC+CCRT+AC group vs. IC+CCRT group** | 1.581 (0.934-2.678) | 0.088 |
| **CCRT+AC group vs. IC+CCRT+AC group** | 1.024 (0.619-1.694) | 0.927 |
| **Overall stage (IVa vs. III)** | 1.233 (0.777-1.958) | 0.374 |
| **Overall stage (IVb vs. III)** | 1.662 (0.907-3.049) | 0.100 |
| **Overall stage (IVa vs. IVb)** | 0.742 (0.414-1.329) | 0.315 |
| **EBV-DNA levels (≥1500 vs.<1500))** | 1.955 (1.263-3.027) | 0.003 |
| **Smoking (Yes vs. No)** | 1.222 (0.773-1.933) | 0.391 |

*P* value was calculated by Cox proportional hazards model; CI: confidential interval.
